# Supplementary figures and images for: Fisher linear discriminant analysis for classification and prediction of genomic susceptibility to stomach and colorectal cancers based on six STR loci in a northern Chinese Han population
Source: PeerJ. 2019 May 28;7:e7004. doi: 10.7717/peerj.7004 (PMC6544021; doi:10.7717/peerj.7004)

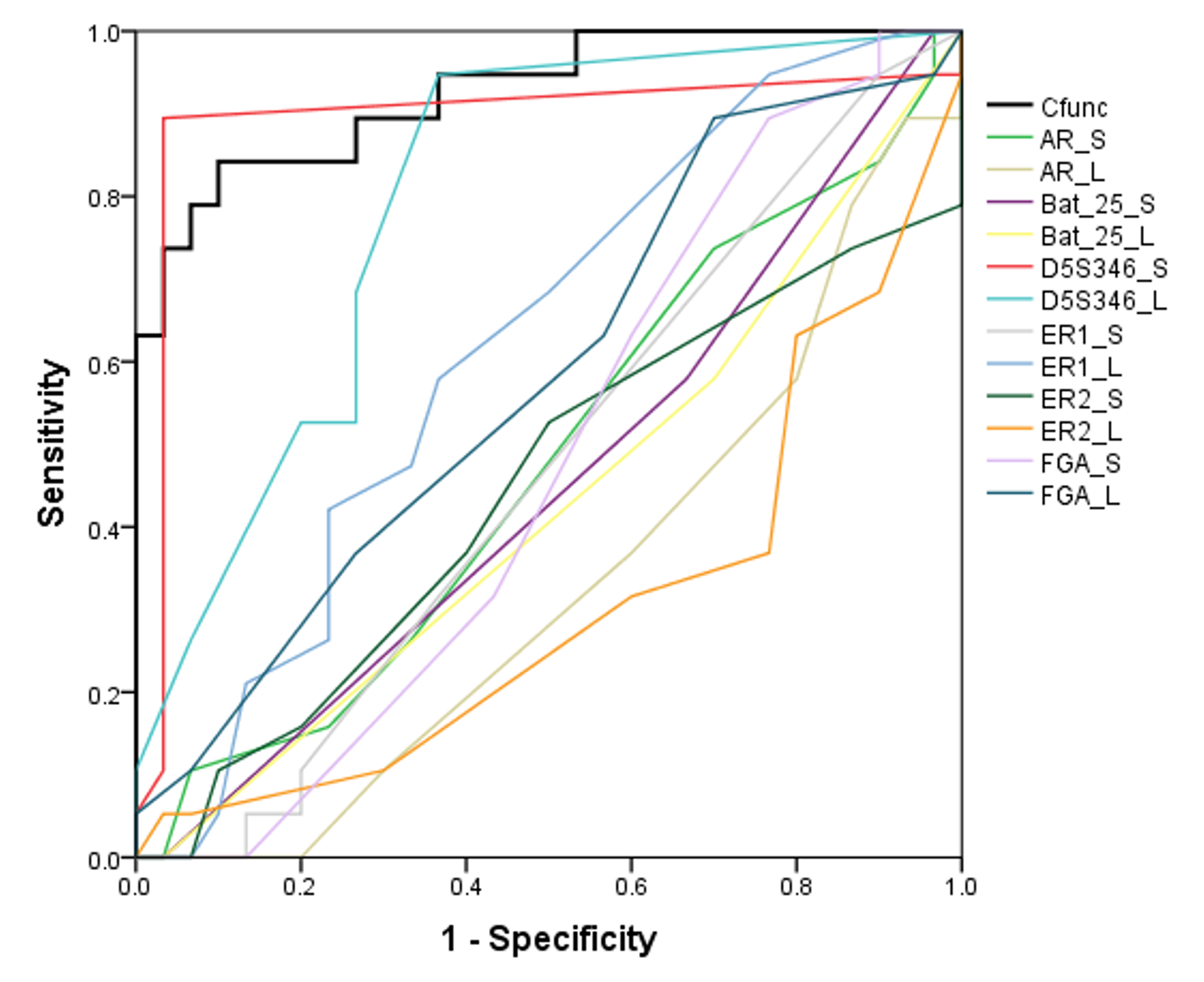

Supplement: Figure S1 [file peerj-07-7004-s001.png]

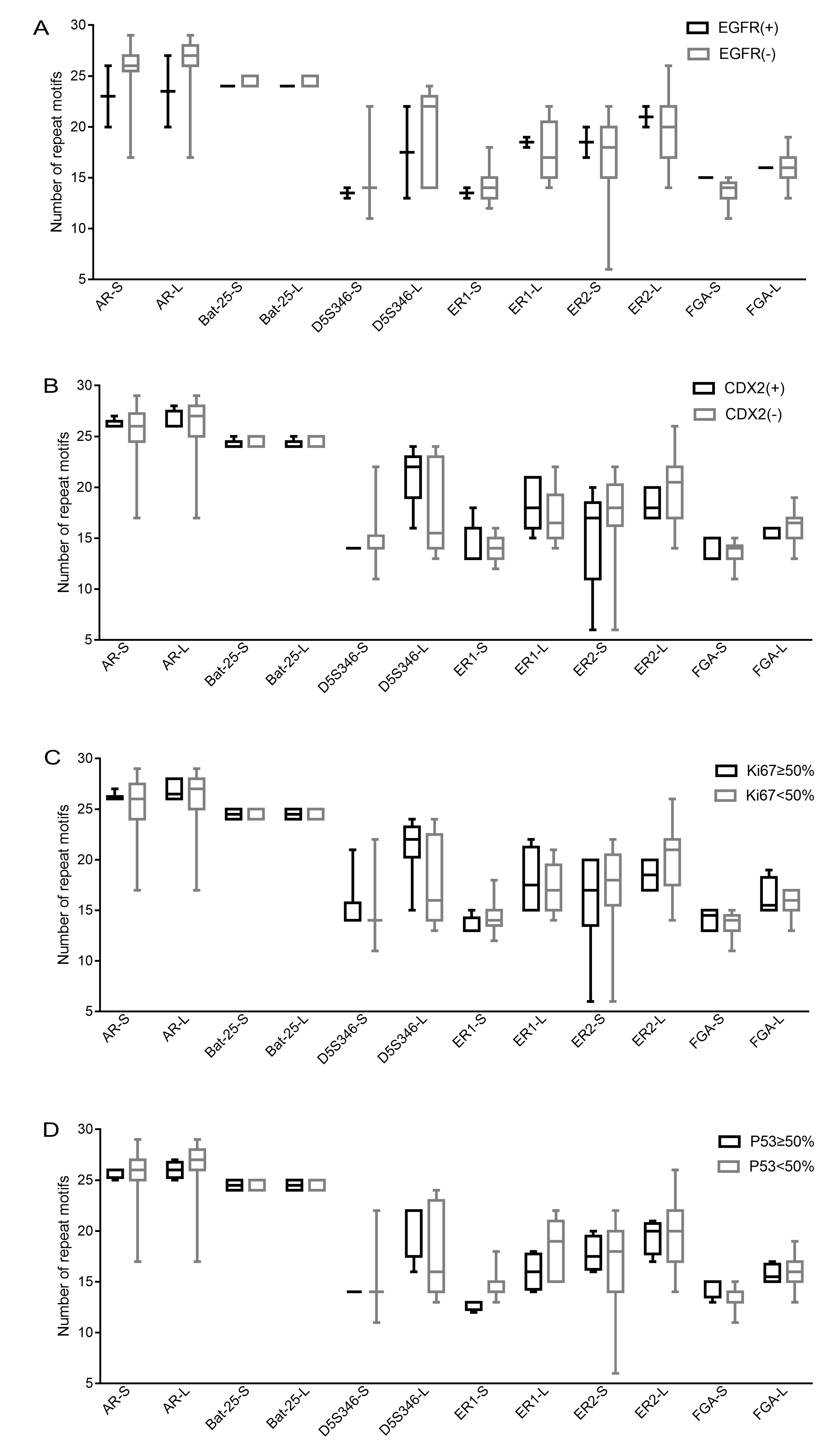

Supplement: Figure S2 — Copy number of STR loci between EGFR (–) and (+) groups (A), CDX2 (–) and (+) groups (B), low and high Ki67 expression groups (C), and low and high P53 expression groups (D). [file peerj-07-7004-s002.png]

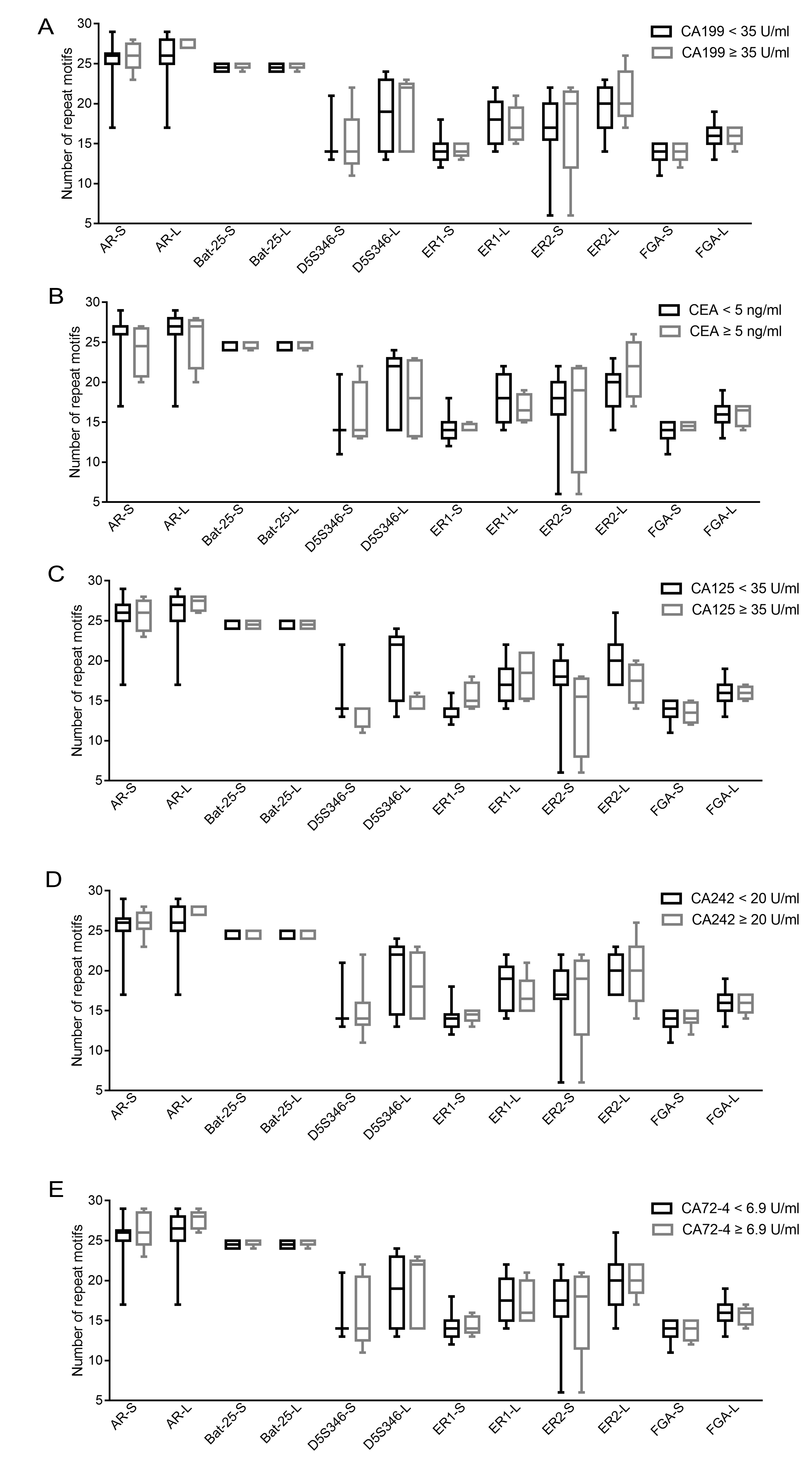

Supplement: Figure S3 — Copy number of STR loci between normal and abnormal CA199 groups (A), normal and abnormal CEA groups (B), normal and abnormal CA125 groups (C), normal and abnormal CA242 groups (D), and normal and abnormal CA72-4 groups (E). [file peerj-07-7004-s003.png]
